# ENGINE—An EHS Project for Future Guidelines

**DOI:** 10.3389/jaws.2024.13007

**Published:** 2024-07-12

**Authors:** Cesare Stabilini, Stavros Antoniou, Frederik Berrevoet, Marja Boermeester, Umberto Bracale, Andrew de Beaux, Barbora East, Hakan Gök, Manuel Lopez Cano, Filip Muysoms, Sara Capoccia Giovannini, Maarten Simons

**Affiliations:** ^1^ Department of Integrated Surgical and Diagnostic Sciences, IRCCS Ospedale Policlinico San Martino, University of Genoa, Genoa, Italy; ^2^ Department of Surgery, Papageorgiou General Hospital, Thessaloniki, Greece; ^3^ Department of General and Hepatobiliary Surgery and Liver Transplantation Service, University Hospital Medical School, Ghent, Belgium; ^4^ Amsterdam UMC, Department of Surgery, University of Amsterdam, Amsterdam, Netherlands; ^5^ Department of Medicine, Surgery and Dentistry, University of Salerno, Salerno, Italy; ^6^ Spire Murrayfield Hospital, Edinburgh, United Kingdom; ^7^ 3rd Department of Surgery, 1st Medical Faculty of Charles University, Motol University Hospital, Prague, Czechia; ^8^ Hernia Istanbul^®^ , Comprehensive Hernia Center, Istanbul, Türkiye; ^9^ Abdominal Wall Surgery Unit, University Hospital Vall d’Hebrón, Barcelona, Universidad Autónoma de Barcelona (UAB), Barcelona, Spain; ^10^ Abdominal Wall Surgery, AZ Maria Middelares, Ghent, Belgium; ^11^ Department of Surgery OLVG Hospital Amsterdam, Amsterdam, Netherlands

**Keywords:** hernia, guidelines, GRADE method, recommandations, methodology

## Abstract

Clinical guidelines are evidence-based recommendations developed by healthcare organizations or expert panels to assist healthcare providers and patients in making appropriate and reliable decisions regarding specific health conditions, aiming to enhance the quality of healthcare by promoting best practices, reducing variations in care, and at the same time, allowing tailored clinical decision-making. European Hernia Society (EHS) guidelines aim to provide surgeons a reliable set of answers to their pertinent clinical questions and a tool to base their activity as experts in the management of abdominal wall defects. The traditional approach to guideline production is based on gathering key opinion leader in a particular field, to address a number of key questions, appraising papers, presenting evidence and produce final recommendations based on the literature and consensus. The Grading of Recommendations Assessment, Development and Evaluation (GRADE) method offers a transparent and structured process for developing and presenting evidence summaries and for carrying out the steps involved in developing recommendations. Its main strength lies in guiding complex judgments that balance the need for simplicity with the requirement for complete and transparent consideration of all important issues. EHS guidelines are of overall good quality but the application of GRADE method, began with EHS guidelines on open abdomen, and the increasing adherence to the process, has greatly improved the reliability of our guidelines. Currently, the need to application of this methodology and the creation of stable and dedicated group of researchers interested in following GRADE in the production of guidelines has been outlined in the literature. Considering that the production of clinical guidelines is a complex process, this paper aim to highlights the primary features of guideline production, GRADE methodology, the challenges associated with their adoption in the field of hernia surgery and the project of the EHS to establish a stable guidelines committee to provide technical and methodological support in update of previously published guideline or the creation of new ones.

## Introduction

Clinical guidelines are evidence-based recommendations developed by healthcare organizations, professional societies, or expert panels to assist healthcare providers and patients in making appropriate and reliable decisions regarding screening, diagnosis, treatment, and follow-up care for specific health conditions. Clinical guidelines aim to enhance the quality of healthcare by promoting best practices, reducing variations in care, and at the same time, allowing individualised or tailored clinical decision-making. They are typically updated regularly to incorporate new research findings and changes in clinical practice. Healthcare providers may use clinical guidelines to inform their decisions, while patients may use them to gain a better understanding of their health conditions and treatment options. The production of clinical guidelines is a complex process that significantly takes up both healthcare professionals and patients’ time.

European Hernia Society (EHS) guidelines aim to provide surgeons who manage abdominal wall defects with a reliable set of answers to their pertinent clinical questions and a tool to base their activity as experts [1, 2].

This paper highlights the primary features of guideline production, GRADE methodologies, and the challenges associated with their adoption in the field of hernia surgery.

## The Project

To establish a stable guidelines committee to provide technical and methodological assistance to the Secretary of Science of the EHS, whenever an update of a key question included in a previously published guideline or the creation of a new one is required.

## Guideline Methodology

After several years of producing guidelines in a more traditional way (top-down approach with poor control on diversity and stakeholder involvement), it has become evident that some of the processes employed in creating clinical guidelines have serious limitations. These range from the system for appraising evidence to the selection and involvement of stakeholders, as well as the consideration of external factors beyond mere evidence that may affect the final guidelines. All of these limitations have resulted in heterogeneous and sometimes non-transparent approaches to guideline development, confusion to the reader and as a possible consequence leading to limited adoption in everyday practice.

The Grading of Recommendations Assessment, Development and Evaluation (GRADE) method offers a transparent and structured process for developing and presenting evidence summaries, as well as for carrying out the steps involved in developing recommendations. Its main strength lies in guiding complex judgments that balance the need for simplicity with the requirement for complete and transparent consideration of all important issues.

The GRADE methodology [3] was developed in the early 2000s by an international group of researchers and methodologists who aimed to enhance the quality and transparency of healthcare recommendations. The initial version of the GRADE approach was published in 2004 [4], and since then, it has undergone several updates and revisions to incorporate new evidence and feedback from users. Today, the GRADE approach is widely recognized as a leading method for assessing the quality of evidence and making recommendations in healthcare research.

A common misconception is related to the essence of the GRADE methodology. GRADE has two key components: appraisal of the scientific evidence (i.e., how confident we are about the beneficial and harmful effects of an intervention), but also a solid framework to place the scientific evidence in the context of developing recommendations (i.e., the so-called Evidence-to-Decision framework – EtD).

The key points of GRADE that will be incorporated into EHS guideline development are as follows:

1) Identifying meaningful clinical questions (Key Question – KQ) is a prerequisite for starting the entire process. This step has to include all possible stakeholders including patients.
2) Conducting a *de novo* systematic review (SR) to gather and evaluate evidence on the topic, (although updating a recent SR assessed to be of good methodological quality is also an option).
3) Assessing the certainty of the evidence across all the outcomes prioritized and deemed critical for judgement.
4) Gathering all evidence related to patients’ values, resources used, acceptability, feasibility, and equity of the interventions being evaluated.
5) Preparing recommendations, taking in account all the implications of the recommendations and the adoption of the interventions.

### Challenges in Hernia Surgery

The traditional approach to guideline production in the EHS involved gathering key opinion leaders in a particular field to address a number of key questions. These experts, mixed with young researchers, were responsible for appraising papers and presenting evidence. Final recommendations and suggestions were developed based on the literature and discussed face-to-face among all experts to reach a consensus. While EHS guidelines were of overall good quality, the application of a formal evidence-to-decision framework (EtD) with a structured, transparent, and reliable process has been infrequently adopted. Nevertheless, the EHS has always encouraged discussion that considered various aspects of recommendations (applicability, patients’ values, etc.,) and the final effect on stakeholders, including the surgical audience. The shift towards GRADE implementation began with EHS guidelines on open abdomen [5], with increasing adherence to the process and the involvement of Cochrane experts and certified GRADE methodologists. This has greatly improved the reliability of our guidelines.

Currently, the need to move forward with this methodology has been outlined in the literature [6] and during several executive board meetings. To satisfy this need, a stable and dedicated group of researchers interested in following this methodology should be established supported where needed by experts in Guideline Methodology.

## The Recommendations—What Is Strong and What Is Weak?

Since the 1970s, an increasing number of organisations in the medical field have used various systems to grade the quality (level) of evidence and the strength of recommendations. Unfortunately, organisations use different systems to categorize their guidance, which means that evidence can be considered differently (II-2, B; C+, 1; or strong evidence) depending on which system is used, leading to confusion and ineffective communication among clinicians and patients. Despite attempts to label recommendations with synthetic alpha-numerical tags, the varying systems create obstacles for clear communication.

The GRADE system aims to produce recommendations that are logical and easily understood. It uses four levels (high, moderate, low, very low) to describe the certainty of evidence and two levels (weak/conditional; strong) to indicate the strength of a recommendation [3]. In principle, a high or moderate level of certainty will result in a strong recommendation, provided there are no important associated adverse effects and no substantial variability in patients’ values and preferences is anticipated. A low or very low level of certainty will generate a weak or conditional recommendation either in favour or against a particular treatment; these levels cannot generate a strong recommendation solely on the evidence. However, it is within the panel´s power to upgrade or downgrade the strength of the recommendation based on expert opinion (check the Certainty of Evidence section), with justification and in exceptional cases. For example, an all-or-none effect, such as providing resuscitation in sepsis, in which case there are detrimental effects if not provided, even if there is no evidence from randomized studies.

End-users of the guideline usually accept the concept of “strong recommendations” because they are considered reliable and compelling. These recommendations come from high level of evidence and are supposed to be recommendations for treatments in which positive effects clearly outweigh the possible negatives under almost every circumstance. In particular, a strong recommendation means that most patients would ask for the treatment, healthcare professionals should provide it with appropriate skill levels (with explanation on the reasons for not offering it, if applicable), and policymakers should ensure that such a treatment is available.

The term “weak recommendation” causes more problems in comprehension. End-users often feel dissatisfied with the weak recommendation because they desire qualified instructions for applying the best treatment to a particular case. Moreover, sometimes the word “weak” implies unreliable information that can be avoided, ignored, or violated. However, the GRADE method has a different concept for weak recommendations. This type of wording of such recommendation expresses that, for a particular treatment, a further process of shared decision making with the patients and/or relatives is the best way to approach the problem [7], including the analysis of alternatives. For these reasons the term “**conditional recommendation**” is more appropriate to indicate that a treatment could be the best under certain conditions and from certain point of evaluation.

### Challenges in Hernia Surgery

Apart from the initial version of inguinal hernia guidelines in 2009 [8], the EHS has adopted GRADE terminology since the beginning, recognising its value and effectiveness, although it has been a journey to adopt all the elements of GRADE.

If we examine the “*strong recommendations”* in EHS guidelines, it is interesting to note that minimally-invasive treatment for inguinal hernia repair received a strong recommendation for its clear advantages in treating bilateral and recurrent cases [8]. However, if we consider limiting factors such as technical equipment, patient frailty, and surgeons proficiency, we can understand that a weak recommendation could also be valid, especially in developing countries and where/when surgeons may not be adequately trained in minimally invasive surgery, despite a large body of evidence favouring this approach [9]. In such situations, a conditional or weak recommendation could assist clinicians in making shared decisions when minimally invasive surgery is not available [10]. Conversely, a strong recommendation could be considered if the panel wishes to emphasise the beneficial effects of minimally invasive surgery and encourage its use for patients in the environment where laparoscopy is underutilised [9, 11]. Accepting that not all patients will be suitable for such an approach.

Regarding “*weak recommendations,”* the guidelines on the management of open and burst abdomen [5], relying primarily on expert guidance, conform most closely to GRADE standards among all published EHS GLs. Despite being based on very low certainty of evidence, they have achieved their goal of providing information for critical clinical scenarios and being accepted in the surgical community, showing a practical influence in research planning as well.

## Certainty of Evidence and Critical Appraisal of Literature

In GRADE, literature appraisal requires the creation of a formal systematic review for each of the KQs in the guideline. The clinical question is formulated according to the traditional PICO scheme: Patients (the clinical subset of individuals affected by a specific disease), Intervention (the treatment under scrutiny), Control (the standard or alternative treatment) and Outcome (the type and extent of effect that the treatments have on the disease). A formal search, conforming to PRISMA requirements, is performed in at least two databases (Pubmed, SCOPUS, Cochrane, etc).

Pertaining Grey literature, inclusion of abstract data in systematic reviews has some serious concerns. Abstracts of primary research provide concise information on a study’s purpose, methods, main results, and conclusions but lack detail for critical appraisal of evidence. However, the information presented in conference abstracts is highly variable in reliability, accuracy, and level of detail. Importantly, abstracts are frequently inconsistent with full reports [12]. Indeed, since risk of bias assessment is not feasible when the full study report is not available, the standard judgement is usually that the study is at high risk of bias. This study can typically be included in the summary analysis. If, in a sensitivity analysis, the summary effect estimates of high versus low risk of bias studies are similar, the overall effect estimate can be considered without downgrading the certainty of the evidence for risk of bias. An exception is when, for a study that provides its results in the form of an abstract, the study protocol is available. In such case, the information provided in the abstract occasionally allows for full risk of bias assessment.

Panellists rate the outcomes to be registered and used to produce the judgment among all possible outcomes (general complications, surgical outcomes, disease-specific, patient-reported) using voting (usually anonymous online survey). Outcomes are voted using a combined categorical and numerical rating system: critical (7–9), important (6-4), low relevance (3-1). No more than seven outcomes can be categorized for each KQ. Subsequent voting determines the minimum significant differences among treatments for the analysed outcomes, allowing panellists to define which are clinically relevant for expressing the judgement. For instance, a difference of 5‰ in the incidence of wound infection might not be important to direct the direction of a recommendation towards the intervention versus a comparator.

As previously discussed, evidence can be graded in high, moderate, low, or very low. In the context of a systematic review, the quality ratings reflect our confidence that the estimates of the effect are correct. In the context of producing recommendations, quality ratings reflect our confidence that the estimates of an effect are adequate to support a particular decision or recommendation [13]. GRADE does provide a reproducible and transparent framework for grading certainty of evidence [14]. Evidence from randomised trials begins with high certainty and can be downgraded based on specific criteria. Evidence from non-randomised studies (NRS) not only in hernia papers but also in other speciality literature [15] starts with a low level of certainty due to their inherent methodological inferiority, but may be upgraded.

Caution needs to be taken to base an upgrade or downgrade on solid grounds without intellectual bias. The criteria for upgrading and downgrading a body of evidence relate to eight items which are discussed further below.

## Reasons for Downgrading Evidence

1. **Risk of bias:** The reliability of a study’s results may be compromised due to limitations in its design or conduct, known as bias. It can be challenging to determine the degree of potential bias(s) that can affect the results, and thus lead to reduced confidence in the estimated effect of the trial. There are multiple tools available to evaluate the risk of bias in individual randomised trials and observational studies. Currently, the standard tool for RCTs is RoB2 [16], and for NRS, it is ROBINS-I [17]. GRADE is used to rate the body of evidence at the outcome level rather than the study level. Authors must also make a judgement regarding whether the risk of bias in the individual studies is significant enough to lower their confidence in the estimated treatment effect.
2. **Imprecision**: The rating for imprecision focuses on the 95% confidence interval around the best estimate of the absolute effect [18]. Confidence intervals refer to the range in which the true estimate of effect of a treatment plausibly lies. When considering the quality of evidence, the question is whether the CI around the estimate of treatment effect is narrow enough to support a consistent clinical decision if the true effect was at the upper versus the lower end of the CI. Authors may also rate down for imprecision if the effect estimate comes from only one or two small studies or if there was only an overall small sample size of patients in the available evidence.
3. **Inconsistency**: Certainty in a body of evidence is highest when the effects across selected studies are consistent. When rating for inconsistency, authors should evaluate consistency of point estimates, overlap of CIs, as well as statistical or conceptual heterogeneity [19].
4. **Indirectness**: Evidence is most certain when studies directly compare the interventions of interest in the population of interest and report the outcome(s) critical for decision making. Certainty may be rated down when these criteria are not met [20].
5. **Publication bias:** Publication bias is one of GRADE’s subtlest domains, requiring making speculations about missing evidence. Various statistical and visual methods, such as Eggers’s test and funnel plot, are used to detect publication bias. Publication bias is more common with observational data and when most of the published studies are funded by industry [21].

## Reasons for Upgrading Evidence

6. **Large magnitude of effect**: Upgrading the level of evidence can be justified when there is a clear and significant effect of a treatment, even if the evidence comes from observational or time series studies. Confounding alone is unlikely to explain associations with a relative risk (RR) greater than 2 (or less than 0.5), and it is very unlikely to explain associations with an RR greater than 5 (or less than 0.2). Therefore, the GRADE group recommends upgrading the quality of evidence by one category (usually from low to moderate) for associations greater than 2 RR, and by two categories for associations greater than 5. Upgrading is usually considered when the studies have low risk of bias, except for randomisation [22].
7. **Dose Response Gradient**: The presence of a dose-response gradient strongly supports the assumption of a cause-effect relationship. Such a gradient may increase our confidence in the findings of observational studies and thus enhance the assigned quality of evidence [22].
8. **All Residual Confounding Would Decrease Magnitude of Effect**: On occasion, all plausible confounders and biases from observational studies unaccounted for in the adjusted analysis (i.e., all residual confounders) of a rigorous observational study would result in an underestimate of an apparent treatment effect. This typically occurs when an experimental treatment is administered only in frail subjects, yet they still experience better outcomes. In such cases, it is likely that the actual intervention effect is even larger than the data suggest.

The analysis of the certainty of evidence is usually performed using several online tools, presented through GRADEpro GDT software. The summary of findings (SoF) table is created for each KQ. SoF tables are documents in which the evidence and the effects of the comparators are expressed for every critical outcome considered.

### Challenges in Hernia Surgery

Table 1 presents examples of upgrading and downgrading the certainty of evidence observed in the literature on hernia surgery that has already been published. The body of evidence in hernia surgery has unique features that make it different from other surgical specialities.

**TABLE 1:** GRADE items and the examples of upgrading and downgrading the certainty of evidence observed in the literature on hernia surgery.

GRADE item	Examples in literature	Graphical representation
Risk of bias	A retrospective series of 235 heterogeneous non-consecutive patients operated in a third-referral center with bridged repair for difficult incisional hernia with 3 different mesh type	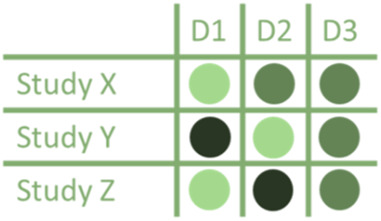
Inconsistency	Prophylactic Antibiotic effect on superficial surgical site infections in open inguinal hernia repair	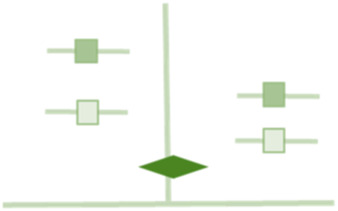
	*Tian XJ, Int Wound J. 2023 Apr;20(4):1191–1204*	
Imprecision	Surgical site occurrences in robotic TAR vs. open TAR	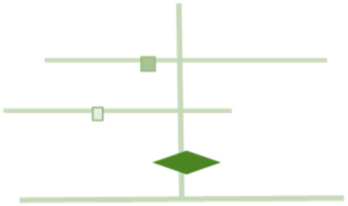
	*Bracale U, Hernia. 2021 Dec;25(6):1471–1480*	
Indirectness	Midline restoration in open incisional hernia repair recommended on the basis of a positive effect in QoL during IPOM repair	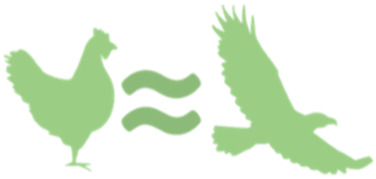
	*Bernardi K, Ann Surg. 2020 Mar;271(3):434–439*	
Publication bias	The comparison between sublay and onlay hernia repairs	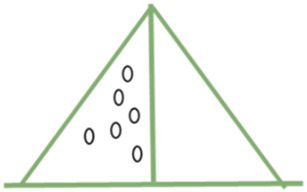
	*Beckers Perletti L, Hernia. 2022 Feb;26(1):3–15*	
Large magnitude of effect	The rate of seroma in the comparison between sublay and onlay hernia repair	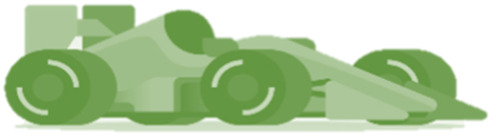
	*Beckers Perletti L, Hernia. 2022 Feb;26(1):3–15*	
Dose response gradient	The effect of obesity on SSOs in abdominal wall reconstruction	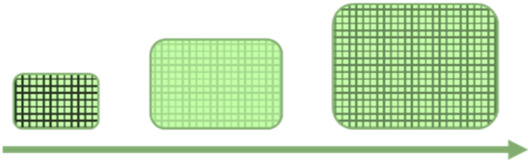
	*Owei et al. Surgery 2017;162:1320–1329*	
All residual confounding would decrease magnitude of effect	The recurrence rate of parastomal hernia treated with Sugarbaker vs. Keyhole repair	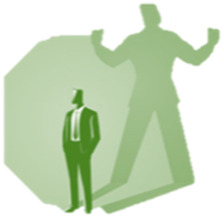
	*Fleming A, J Gastrointest Surg . 2022 Dec 5. doi: 10.1007/s11605-022-05412-y*	

Firstly, abdominal wall hernia research represents one of the more active fields in surgical research with a constantly increasing number of publications in Pubmed, at a rate that surpasses the overall growth rate of the entire database. Secondly, the availability of RCTs is higher in hernia surgery than in other surgical fields, establishing the potential for high quality recommendations. Thirdly, since the early 2000s, the EHS has initiated a process of collaboration and inclusion of international experts to identify and harmonise various research standards. However, biases still exist and seriously impede every iteration of the GL development process. The main examples are reported below.

The treatment of an abdominal wall defect, regardless of whether it is primary ventral, incisional or groin, should improve patients’ Quality of Life and reduce the risk for future acute complications. Despite this concept, the surgical community still focuses on recurrence rates and recurrence free survival, which is actually a long-term complication and could be considered a surrogate outcome. These outcomes are relevant but less informative for a panel than patient-reported outcomes (PROMs). However, the full utilization of PROMs in hernia research is impeded by a lack of consensus in the field. There are no agreed-upon standards for PROM selection, and there are issues regarding PROM validation and poor adherence to PROM reporting guidelines [23–26]. Moreover, having a patient representative in a guideline committee is very valuable.

Another example of important bias lies in the field of non-inguinal hernias, where almost all RCTs providing potential high-level evidence enrol a mixed cohort of primary ventral and incisional cases. This introduces high conceptual heterogeneity and a serious source of bias in the design of the studies. In fact, it has been shown [27, 28] that surgical performances are completely different in the two clinical scenarios, with better results for primary defects, that can mitigate the worse outcomes of incisional hernias and improperly favour one treatment over another.

Finally, the body of evidence on hernia related issues suffers from the same problems encountered in other surgical fields where RCTs can be challenging at all stages of the process including randomisation, patient enrolment and accrual, long term follow up and obviously raising the funds to support such research. Despite these issues, properly conducted and powered RCTs are informative to produce reliable recommendations [29].

## Evidence to Decision Framework

**FIGURE 1:**
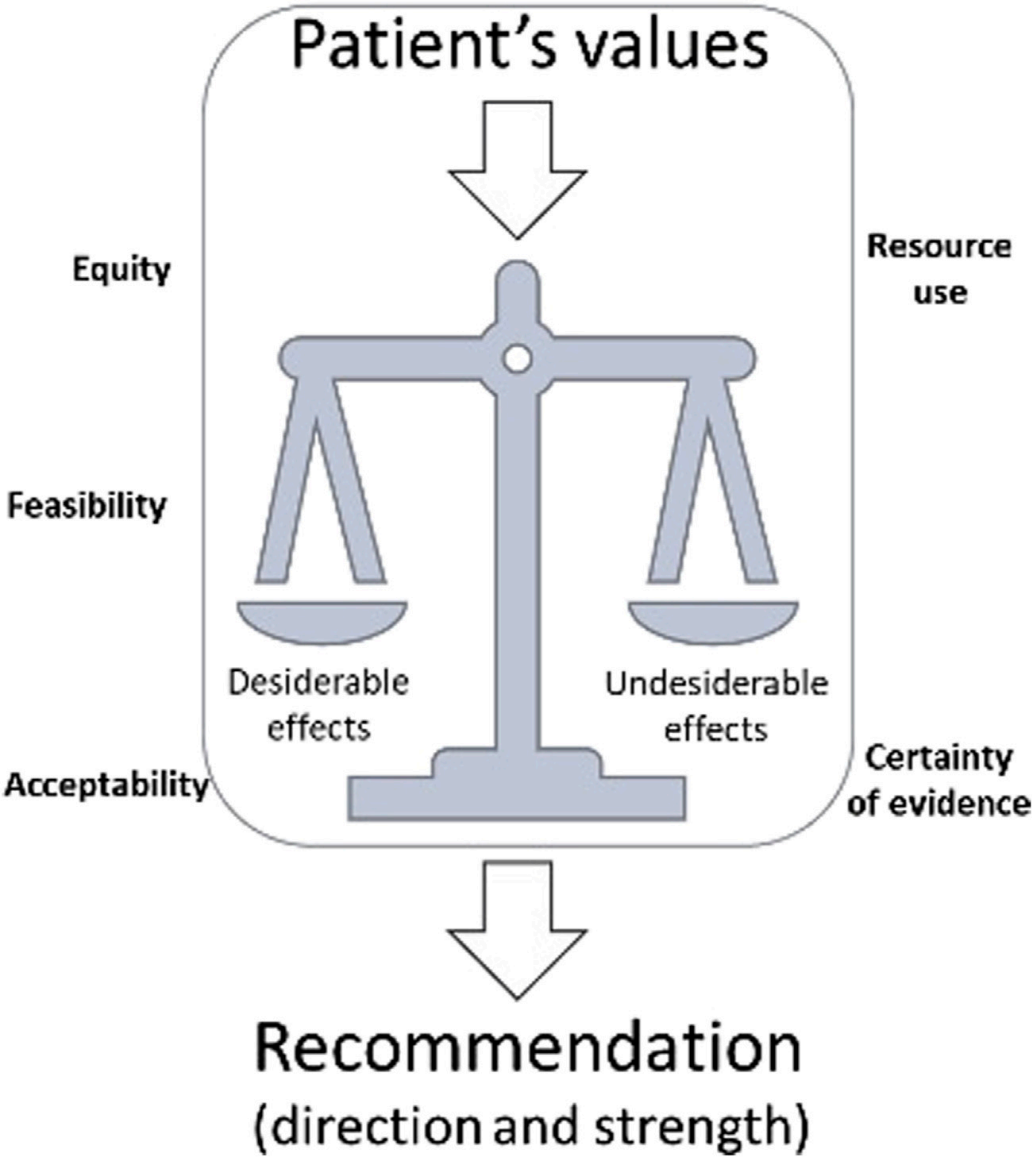
Representation of EtD framework.

Evidence to decision (EtD) framework is the final stage of the guideline production process in which all available evidence is collected, presented and discussed among the panel (Figure 1). The GRADE methodology differs from other methods in that the direction and strength of recommendations are not solely based on evidence from clinical comparisons but instead come from integrating several ancillary parameters that contribute to forming the final judgement. This entire process is tracked and recorded transparently, and while there is still some subjectivity in interpreting all the domains, it is clearly documented and made available for analysis by third parties, other researchers and final users [30].

The parameters used to drive the recommendations are numerous, including the magnitude of the estimates of effect of interventions on important outcomes, confidence in those estimates, estimates of typical values and preferences (confidence in estimates, variability), resource use, acceptability from the different stakeholders, equity, and feasibility.

A key concept of GRADE is the balance of desirable and undesirable effects (also called trade-offs), which group together the magnitude of effect and values and preferences. The desirable (benefit from a treatment) and undesirable effects (side effects) are extracted from the literature in terms of absolute and relative numbers and evaluated in relation to the stakeholder’s point of view (usually the patients’ values and preferences). The larger the gradient between the desirable and undesirable effects, the higher the likelihood that a panel will provide a strong recommendation. However, a major issue in this process is the scarcity of publications exploring the priorities of patients. The involvement of patients or group representatives in the process is thus self-evident, but has been a neglected part of EHS guidelines.

As mentioned, confidence in estimates is an important driver of the recommendation and their strength. When there is high confidence in benefits and low confidence in possible harms (particularly in the long term), the issued recommendation will be weak on a conservative approach. Usually, the more closely balanced the trade-offs between desirable and undesirable outcomes, the more likely that low confidence for any critical outcome will result in a weak recommendation [31]. Economic evaluation is defined as the comparative analysis of alternative interventions in terms of both their costs and effects. There are three main types of economic evaluation in healthcare: cost-effectiveness analysis, cost utility analysis and cost-benefit analysis. Although, the analysis of these different study designs is beyond the scope of this document and will not be discussed, it is relevant to point out that economic evaluation plays an important role in the definition of the recommendation. It should be considered the same way as other clinical outcomes in terms of limitations, magnitude, and certainty. The different economic impact of an intervention over control informs the panel and helps substantiate the decision to adopt or not the treatment from the point of view of the healthcare system.

Acceptability, equity, and feasibility all represent the true impact of the recommendation and a forecast for the adoption of the guideline in practice. Health systems differ greatly across the world, with disparities between developed and developing countries, and differences among countries in the same continent concerning beliefs, religion and culture, which make it impossible to generalise a recommendation to all the possible scenarios. Accordingly, the true aim of GRADE is to “globalise evidence and localise decisions” highlighting what is right in a developed reality and what should be aimed at in developing one. In the past recommendations were frequently judged obscure or arbitrary in their development and far from everyday activity, reducing the final uptake and dissemination of the guidance. This way of producing guidelines is clearly more articulated and demanding than traditional surgical guidelines but actually more effective.

It should be highlighted that each of the EtD domains carry different weights in different contexts. For instance, economic considerations might not be as important in a guideline developed from a patient perspective, or one that is meant to apply in private practice. Conversely, use of resources would be a decisive factor in a guideline that is developed to be applied in developing countries.

### Challenges in Hernia Surgery

The use of an EtD framework as a roadmap for developing recommendation in hernia surgery presents an opportunity and a challenge that will enhance the quality of guidelines and the level of care for our patients. Recommendations that are based only on benefits and harms, at best, derived from a suboptimal body of evidence are no longer sustainable. Hernia disease is the most prevalent condition in general surgery, both in terms of patients and healthcare stakeholders. Therefore, adopting the correct analytical perspective and involving end-users as much as possible would have a significant impact on a large area of interest.

The application of a structured EtD will increasingly encourage the creation of a body of evidence, new definitions, and data around patients’ views, values, and preferences, to inform our panels. As discussed, the evidence around these parameters is scant, heterogeneous, and inconsistent, making it a challenge to correctly interpret the needs of such a vast group of people. Additionally, there is a total absence of data concerning the acceptability, feasibility, and equity of our treatments, which, with technical development, are becoming increasingly relevant. Several examples can be used to clarify the implication of these concepts when applied to hernia surgery, demonstrating how a comprehensive and integrated approach can effectively influence our recommendations in different ways:

- There have been extensive discussions and several randomised, high-quality trials on the use of meshes with different density materials in inguinal hernia repair [32, 33]. However, these studies have focused almost entirely on recurrence rate without being able to define their impact on QoL of the patients, except for a transient short-term benefit in open hernia repair. It is difficult to make a recommendation without input from the patients defining the threshold of significance and whether they value this type of benefit or not. And the picture is further muddied from the fact that qualitative research on patient’s perspectives is difficulty published in high-ranking scientific journals even if its importance is acknowledged almost universally reducing the possibility to shift the attention to PROMs.

Qualitative research informs the domains of patients’ and other stakeholders’ values and preferences, acceptability, feasibility, and equity, of the evidence-to-decision framework. In the absence of qualitative evidence, there are several options. These include surveys (e.g., of EHS members, patient organizations), focus groups (of patients, surgeons, and allied professionals), and/or direct representation of patients through patient partners and other stakeholder groups in the panel. The choice among options will be made depending on the topic and the availability of resources.

- It can be difficult to implement the outcomes of research into general clinical practice, e.g., prevention of incisional hernias. The hernia community has conducted relevant high-quality studies [34, 35] and produced up to date guidelines [36]. Nevertheless, the rate of penetration of small-bites technique and mesh augmentation is low and probably not widely accepted by general surgeons [37]. This highlights the need for dissemination and implementation strategies in the surgical community to increase acceptability among the target population. Understanding such barriers may help find solutions to overcome these. And even if chance is not affected, encourage auditing of outcomes so that surgeons know they results, such as incisional hernia rate.
- New techniques have been introduced into clinical practice by pioneering surgeons to address specific clinical scenarios (eTEP, eMILOS, robotics) as well as new materials to overcome relevant surgical needs (biologic, biosynthetic). Their inclusion in current guidelines would require an unbiased discussion on their impact on resources use, not only in terms of money but also on equity in terms of operative theatre occupation and availability in various health systems, as well as acceptability by all surgeons. Finance is limited in most healthcare systems, and what is spent on one patient cannot be spent on another.

## The Panel

### Patient Involvement

Involving patients in guideline panels is a crucial step to ensure that the perspectives and needs of those who are directly affected by the guidelines are considered. Patients can provide unique insights based on their personal experiences with the condition or treatment in question, as well as their preferences and values. But we should also remember that healthcare workers become ill too, and as hernia is a common condition, many so called experts on a guideline panel may also be current or past patients.

There are several ways to involve patients in guidelines panels. One approach is to include patient representatives as members of the panel. These representatives can be individuals with lived experience of the condition or treatment, or they can be representatives of patient advocacy organisations. Another approach is to gather input from patients through surveys, focus groups, or other forms of patient engagement. It is important to ensure that patients are involved in a meaningful way and that their input is taken seriously. This may require additional resources and training for both patients and guideline developers. Patients may also need support to effectively participate in the guideline development process, such as access to information and resources, and assistance with communication and advocacy. Overall, involving patients in guidelines panels are key to ensuring that guidelines are patient-centred and reflect the diverse needs and perspectives of those who will be directly affected by them.

#### Challenges in Hernia Surgery

Elective hernia repair is the treatment of a benign disease. However as outlined recently from Gram-Hanssen [38], only half of the publications apply patient-reported outcome measures (PROMs) as the primary outcome, and often they are reported using several different methods, which impedes proper evidence synthesis. Actually, the first aim of every abdominal wall defect repair should be aimed at improving patient’s quality of life, while recurrence represents only a possible adjunctive collateral effect of the procedure. In this light, standardisation of PROMs and involvement of patients and their representatives in Guideline development should be aimed at understanding the best treatment of their disease, giving voice to what they consider relevant. Accordingly, EHS board members have facilitated the creation of independent online groups in social media to explore, through online consultations, patients’ attitudes, values, and desires in the field of hernia surgery. East et al. [39] and Jimenez et al [40] showed how patients, along with a sort of underestimation of the gravity of their condition, feel a strong appreciation for a detailed explanation of their operations, the possible adverse events, and want to be involved in a true shared decision-making process with their operating surgeon. Patients are also aware of the condition they are facing, the possible impact on their everyday life, and are more willing, for example, to undergo a more complex intervention than a quick fix one in the pursuit of the definitive treatment while minimizing the risk of recurrence.

Interestingly, both East and Elhage surveys [39, 41] showed around 40% of interviewed patients being concerned of the mesh repair or not happy with their mesh. This can be explained by the fact that hernia surgery is currently facing several negative reports of adverse events that have occurred in the gynae-urological field. Consequently, a number of patients are developing reluctance to the implantation of a permanent synthetic mesh. As a result, an increasing request for tissue repair is seen (mainly in north and central Europe, and North America). If this trend is confirmed on a large scale, it could lead to possible extreme scenarios, for example, a serious downgrade of a recommendation in favour of a mesh due to its reduced acceptability for the patients despite the presence of relevant evidence in favour of improved outcomes.

The other real challenge in hernia surgery is trying to profile those patients who are willing to engage in such a process. Patients who are currently living with a hernia or who have undergone hernia surgery can participate effectively to the guideline panel. Those who are living with a hernia can refer to the impact of the hernia on their quality of life and their preferences for treatment. Patients who have undergone hernia surgery can provide valuable insights based on their personal experiences with the condition and the surgical procedure, even if they have experienced complications or a “poor” outcome. They can provide feedback on the effectiveness of different treatment options, the recovery process, and the impact of surgery on their daily life. A major challenge is represented by the patient with serious complications from the treatment. While their experience is immense in informing about the risk/benefit balance, the personal experience could strongly influence the decision-making process that does not reflect typical outcomes of an intervention. It is necessary to involve a sufficient number of patients to allow them to be comfortable in expressing their opinion and engaging in the process. Recruiting patient representatives to participate in guideline panels can be challenging. A dedicated communication group may increasingly collect experience by understanding better their needs and expectations, thereby increasing participation.

It should be remembered that patients can also have Conflict of Interest issues as discussed next.

### Experts Surgeons and Conflict of Interests (COIs)

The management of conflicts of interest (COIs) in clinical practice guidelines should aim to minimise their real or perceived effects on the final recommendation, and any resulting bias. A recent definition of COI was proposed by Akl et al [42]:

“A COI exists when a past, current, or expected interest creates a significant risk of inappropriately influencing an individual’s judgment, decision, or action when carrying out a specific duty”.

COIs can be categorized at an individual and institutional level, and can be financial, intellectual or personal in nature. COI can arise in several forms. Recent research [43] had demonstrated that a predefined management for COI can have a positive impact on the entire guideline process. In two subsequent iterations of a guideline [44, 45], a more restrictive strategy led to a profound change in the type of recommendations issued. The number of strong recommendations were reduced from 27% to only 5%, with particularly marked reductions in the number of strong recommendations based on low-quality evidence, which fell from 21.5% in the earlier version to only 4.3% in the later version [46]. These findings suggest that panels with careful management of COIs may be less likely to make strong recommendations.

#### Challenges in Hernia Surgery

Abdominal wall surgery, when compared to other general surgical subspecialities, has a high potential for bias due to its frequent use of specialised commercial devices such as meshes, fixation devices, drugs, pre- and postoperative belts, and robotics. This bias is further exacerbated by the various relationships between societies, researchers, and the industry. Additionally, participation in clinical guideline production can contribute to academic career advancement. Finally, being qualified as expert in a field and called to serve on a panel often entails extensive research, writing, and speaking on topics that are the subject of a KQs. By the very nature of being an expert, implies bias. At the same time, guideline production and synthesis of the evidence by non-experts can lead to wrong conclusions. The key is identifying and managing COI in every member of the guideline panel.

Excluding an expert with relevant direct COI from the production of a guideline could compromise the trustworthiness of the recommendations by depriving the panel of important insights into the topic of interest and by being less accurate in the eyes of the stakeholder. Therefore, the EHS acknowledges the possibility and high risk of bias in guidelines due to COIs and proposes the following management strategy:

At the beginning of the guideline production process, all participants must disclose any possible COIs to the Advisory Board of Science. Indeed, it is perhaps better to call them Declarations of Interest, and let the Advisory Board assess them as a conflict or not.

The Advisory Board evaluates the presence or absence of COI based on the following criteria [42]

A. Relevance of the interest to the duty the individual is assuming (high vs. low).
B. Nature of the interest (even higher when indirect).
C. Magnitude of the interest (higher for higher amounts of benefit).
D. Recency of the interest (the more recent, the higher).
  • A member who scores high in at least 3 out of 4 criteria (with one being relevance) is high risk.
  • A member who scores high in 2 items (with one being relevance) is considered moderate risk.

The following restrictions apply:

• Researchers at high risk of COIs cannot become the chair of a guideline to reduce the risk of producing a less credible guideline.
• Researchers at moderate to high risk of COIs cannot vote in a KQ where this risk could be present and should be allocated to different KQs.
• Researchers at moderate risk of COIs can participate in critical appraisal of the literature.
• Researchers with a high risk of COIs can be used as advisors in the context of a KQ at risk.

## Activities of the EHS Guideline Review Committee

The committee serves as a methodological aid to the Secretary of Science and Science Wing for the revision of guidelines or relevant KQs. Guidelines should be updated every 3 years or whenever new evidence emerges in the literature. Guideline updating can be done by selecting KQs with new recent evidence that has the potential to change the recommendation or the certainty of the recommendation, so-called modular updating. Modular updating - instead of complete guideline updating - has the advantage that the process takes less time and thereby changes in evidence and associated recommendations can be done timely.

The management of COI is part of the GRADE methodology and this applies also to the committee itself, but it should not hinder its activity by impeding experts to give their valuable contributions, even if the avoidance of any bias is the clear prerequisite of a trustworthy guideline. The role of the committee in the GDG is providing the needed expert to overview the correctness of the entire process. He or she has no right to vote the recommendation since the final decisions is taken by a panel free of direct COIs preventing their wellknown effect.

The committee is supposed to meet twice a year: one event during the Annual EHS Congress, the second during the year to plan future tasks. The group is composed of certified methodologists and surgeons that will be part of guidelines development and members responsible for updates. This way should allow for a better interface between surgical and methodological needs Currently, the choice of the members is made on two main features: experience in previous guidelines, in particular those made with GRADE methodology, and INGUIDE level 2 certification. The two conditions are relevant since the people involved in the previous document are an optimal asset for prioritizing future updates, on the other side the INGUIDE certification allows to be able to conduct a panel through the process of guidelines development.

In future the EHS is planning to recruit young researcher to have a natural rotation of roles and new forces joining the committee while gaining experience through certification and active participation to guideline development.

In light of the relevance of EHS guidelines as international documents that can be adopted out of the European boundary, the EHS has involved several experts in guidelines from non-european-countries.

### Composition

The committee should consist of at least two members with at least 2nd level GIN certification of a GUIDELINES METHODOLOGIST.

### Activities

a) The committee should monitor the body of evidence in the literature in a systematic and reproducible way on a yearly basis.
  • Conduct scoping reviews with the involvement of young members selected through a call for interest and CV presentation.
  • Prepare a report for upcoming GLs and single KQs requiring review/revision to be sent to the Science Secretary.
b) Select panellists and check for COI.
  • Prepare a report for the Science Wing.
  • Involve patient representatives.
c) Gather data on patient values and preferences in collaboration with Social Media Secretary, the General Secretary, and the Patient Advisory wing of the EHS.
  • Prepare surveys with patient representatives to evaluate their values in relations to specific KQs under preparation or update.
  • Prepare surveys for surgeons to monitor the clinical impact of published guidelines.
d) Assist the Chair in the process of guidelines development.
  • Formulate KQs.
  • Draft protocols.
  • Prepare SRs and meta-analyses with SoF tables in association with GRADE experts whenever available.
  • Prepare EtD.
e) Participate in dissemination and implementation projects of developed guidelines in collaboration with the Board and members of the EHS, and other surgical associations where applicable.
  • Prepare plain language summaries for patients. Together with the Associated and Affiliated Chapter Secretary of the EHS, prepare translations of all guidelines in the languages spoken around Europe including the plain language summaries.
  • Prepare a publication strategy for each guideline.
  • Prepare a long-term strategy of KQs/guidelines that need updating.

## Data Availability

The original contributions presented in the study are included in the article/supplementary material, further inquiries can be directed to the corresponding author.
